# Serum lipid profiles in patients with beta-thalassemia major and intermedia in southern Iran

**Published:** 2010

**Authors:** Sezaneh Haghpanah, Maryam Davani, Behrang Samadi, Afsaneh Ashrafi, Mehran Karimi

**Affiliations:** a Hematology Research Center, Shiraz University of Medical Sciences, Shiraz, Iran

**Keywords:** Beta-Thalassemia Major, Beta-Thalassemia Intermedia, Total Cholesterol, LDL-Cholesterol, HDL-Cholesterol, Triglycerides

## Abstract

**BACKGROUND:**

Beta-thalassemia is considered to be the most frequent hereditary blood disorder worldwide. Lipid abnormalities have been detected in different types of beta-thalassemia. The aim of this study is to assess the lipid profiles in beta-thalassemia major (BTM) and beta-thalassemia intermedia (BTI) patients in southern Iran.

**METHODS:**

The study group consisted of 55 BTM patients and 50 BTI patients. The control group included 130 sex-and age-matched healthy participants. Serum lipids profiles (total cholesterol, triglycerides, LDL-cholesterol, and HDL-cholesterol) as well as hemoglobin (Hb) and ferritin, were compared between the three groups. P value < 0.05 was considered statistically significant.

**RESULTS:**

There were no significant differences between BTM and BTI patients regarding age or sex. Mean triglyceride concentration was not significantly different between patients and controls. Total cholesterol and LDL-cholesterol were significantly lower in patients with BTM and BTI in comparison with controls (p < 0.001). HDL-cholesterol was significantly lower in patients with BTI than in controls (p < 0.03).

**CONCLUSIONS:**

In patients with BTM and BTI, total cholesterol and LDL-cholesterol were lower than in control participants. The mechanisms that may account for these findings are increased erythropoiesis and cholesterol consumption in BTI, and iron overload and oxidative stress in BTM.

Beta thalassemia is considered to be the most frequent hereditary blood disorder worldwide. Iran is located in the thalassemic belt, and higher prevalences (about 10%) are detected in the northern and southern provinces bordering the Caspian Sea and Persian Gulf.^1^–^4^

Lipid abnormalities have been detected in different types of beta thalassemia, and also in various hematological disorders including sickle cell disease, glucose-6-phosphate dehydrogenase (G6PD) deficiency, spherocytosis, aplastic anemia and myelodysplastic syndrome.^5^–^9^ The pathogenesis of these abnormalities is not exactly clear, but there are many suggested mechanisms including plasma dilution due to anemia, accelerated erythropoiesis resulting in increased cholesterol uptake by macrophages and histiocytes of the reticuloen-dothelial system, defective liver functioning due to iron overload, macrophage system activation with cytokine release, and hormonal disturbances.^7^,^10^–^15^

No earlier studies of lipid abnormalities in thalassemic partients in Iran were found. Awareness of physicians to these abnormalities is helpful to avoid unnecessary work up in these patients. The aim of this research is to assess the lipid profiles in two groups of patients with BTM and BTI in southern Iran, and to compare the findings to those in healthy control participants.

## Methods

### 

#### Patients

The study group consisted of patients with thalassemia major (n = 55) and thalassemia intermedia (n = 50) who were selected randomly from the patients referred to the thalassemia clinic of Shiraz University of Medical Sciences, a referral center for thalassemic patients in southern Iran, during 2007 and 2008.

Patients with thalassemia major were diagnosed by clinical history, requirement for regular blood transfusions, and laboratory tests including complete blood count (CBC) and hemoglobin electrophoresis. Patients with thalas-semia intermedia were diagnosed based on medical history, long intervals between transfusions or no need to blood transfusions, as well as laboratory tests including CBC and hemoglobin electrophoresis. Exclusion criteria were having diabetes mellitus, hypothyroidism, hyperthyroidism, renal failure and hereditary hyperlipidemia.

The control group consisted of 130 healthy participants matched for sex and age distribution. They selected from the individuals who referred for check up. Mean age of participants in all three groups together was 22.5 ± 7.3 years (range: 16 to 30 years).

The research protocol was approved by the ethics committee of Shiraz University of Medical Sciences and informed written consent was taken from all patients and controls.

#### Laboratory Methods

Serum lipid profile was determined in all patients and controls following a 12-hour overnight fasting. Total cholesterol (TC), triglycerides (TG) and HDL-cholesterol concentrations were measured with an Alcyon 300Abbott analyzer (USA). LDL-cholesterol concentrations were determined by Friedewald’s formula.^16^ Hemoglobin and ferritin concentrations determined in patients with thalassemia with a Sysmex KX 21 analyzer (Japan) and by radioimmunoassay, respectively.

#### Statistical Analysis

Statistical analysis was done with SPSS software version 15 (SPSS Inc, Chicago IL). Differences between mean lipid values were compared by analysis of variance (ANOVA) and multiple comparisons were done using post-hoc Tukey tests. Age, ferritin and hemoglobin in BTM and BTI patients were compared with Student’s t test. Sex distribution was compared between groups with the chi squared test. P values < 0.05 were considered statistically significant.

## Results

The demographic and laboratory characteristics of patients with thalassemia are shown in table 1. There were no significant differences between BTM and BTI patients regarding age or sex. Mean values of Hb and ferritin in BTM patients were significantly higher than BTI patients (p < 0.01 and p <0.0001, respectively).

**Table 1:** Demographic and laboratory characteristics of thalassemia patients (values other than sex are reported as mean ± SD)

	Thalassemia major (n = 55)	Thalassemia intermedia (n = 50)	P value
Age (year)	22.7 ± 6.1	22.1 ± 6.3	NS*
Sex (M/F)	27/28	27/23	NS
Hb (g/dL)	9.6 ± 1.02	8.88 ± 1.31	0.01
Ferritin (ng/dL)	2660 ± 1990	1036 ± 1417	0.0001

* NS: not significant

As shown in table 2, mean concentrations of triglycerides were higher in patients with BTM and BTI than in the control group, but the difference was not significant (p = 0.091). Total cholesterol and LDL-cholesterol were lower in patients with BTM and BTI in comparison with control participants (p < 0.001); however, there was no significant difference between the patients with BTM and BTI. HDL-cholesterol was lower in patients with BTI than in healthy controls (p < 0.03); however, in patients with BTM difference was not significant compared to the control group.

**Table 2:** Serum lipid profiles in thalassemia patients and healthy subjects (values are reported as mean ± SD)

	Thalassemia major (n = 55)	Thalassemia intermedia (n = 50)	Controls (n = 130)	P value
TG (mg/dL)	110.4 ± 45.3	110.1 ± 92.2	89.9 ± 33.7	0.091
TC (mg/dL)	99.1 ± 22.4	81.1 ± 22.6	147.2 ± 28.6	0.001
LDL-C (mg/dL)	51.3 ± 17.1	34.6 ± 23.9	88.2 ± 23.3	0.001
HDL-C (mg/dL)	33.4 ± 8.3	27.3 ± 9.3	40.3 ± 14.9	0.03

TG: Triglyceride; TC: Total cholesterol; LDL-C: Low-density lipoprotein cholesterol; HDL-C: High-density lipoprotein cholesterol

## Discussion

The aim of this research was to assess the lipid profile in two large groups of patients with BTM and BTI in comparison with a group of healthy individuals. Hypocholesterolemia has been detected in different types of beta thalassemia. The present findings are in agreement with those of Papanastasiuo et al,^11^ Hartman et al,^17^ and Amendola et al,^6^ who showed that TC and LDL-cholesterol levels were lower in persons with BTM and BTI than in the control group, although these values were similar in BTM and BTI patients. In contrast to these results, Ricchi et al^5^ and Friedewald et al^16^ found lower levels of TC and LDL-cholesterol in BTI than BTM.

Lower levels of TC and LDL-cholesterol have been reported in beta-thalassemia minor compared to healthy participants.^12^ Hartman et al^17^ stated that based on general knowledge, an intermediate level of TC and LDL-cholesterol would be expected in patients with BTI, and they suggested that the lower concentrations they found in BTI than BTM patients could be related to the higher level of Hb and ferritin in patients with BTM. Based on the present obtained results, however, TC and LDL-cholesterol were similar in patients with BTM and BTI in spite of the higher Hb and ferritin concentrations in the former group.

Triglyceride levels were similar in patients and control participants, like in Amendola et al^6^ study (who studied BTI only) but different from Hartman et al^17^ and Ricchi et al^5^ studies, who reported that triglycerides were elevated in association with diseases such as thalassemia, probably due to extrahepatic lipolytic activity.

HDL-cholesterol was lower in the present samples with BTI than in the control group, like other reports have noted.^5^,^6^,^17^ This subject could be considered as a predictive value of cardiovascular risk in patients with BTI.

The pathophysiology of hypocholesterolemia is obscure in these hematologic disorders, in which anemia is a common characteristic. The purposed mechanisms include increased erythropoietic activity resulting in increased cholesterol requirements, liver injury due to iron overload, and macrophage system activation with cytokine release.^7^ It seems that the main mechanism of hypocholestrolemia in BTM is severe iron overload and oxidative stress,^17^ but in BTI the major mechanism is accelerated erythropoiesis and enhanced cholesterol consumption.^6^,^17^ The results reported by Ricchi et al^5^ support this idea; they showed lower values of cholesterol in patients with a more severe genotype.

## Conclusions

In conclusion, lower total cholesterol and LDL-cholesterol was found in both BTM and BTI groups compared to healthy control participants, but with no difference between the two thalassemia patient groups. The suggested mechanisms for the decreases in lipids are increased erythropoiesis and cholesterol consumption in BTI, and iron overload and oxidative stress in BTM. Awareness to these findings is helpful to avoid unnecessary evaluation in patients with beta-thalassemia. Based on the present results lower level of HDL-cholesterol in patients with BTI should be a motive for concern of better evaluation of the cardiovascular risk factors in these patients; however more future researches are needed for confirmation and explanation of this relationship as well as clarification of the exact mechanism and clinical consequences of the decreases in lipids in patients with beta-thalassemia.

## Authors’ Contributions

SH performed statistical analysis and edited the manuscript. MD drafted the manuscript and collected data. BS designed the study. AA collected data. MK developed the concept and design of the study and edited the text. All authors have read and approved the content of the manuscript.
